# Distanced self-talk increases rational self-interest

**DOI:** 10.1038/s41598-021-04010-3

**Published:** 2022-01-11

**Authors:** Izzy Gainsburg, Walter J. Sowden, Brittany Drake, Warren Herold, Ethan Kross

**Affiliations:** 1 Psychology Department, University of Michigan, Ann Arbor, USA; 2 Ross School of Business, University of Michigan, Ann Arbor, USA; 3 Department of Behavioral Health, Tripler Army Medical Center, Honolulu, USA; 4 Harvard Kennedy School, Harvard University, Cambridge, MA 02138 USA; 5 University of California, Los Angeles, Los Angeles, USA; 6 University of Arkansas, Fayetteville, USA

**Keywords:** Psychology, Human behaviour

## Abstract

Does stepping back to evaluate a situation from a distanced perspective lead us to be selfish or fair? This question has been of philosophical interest for centuries, and, more recently, the focus of extensive empirical inquiry. Yet, extant research reveals a puzzle: some studies suggest that adopting a distanced perspective will produce more rationally self-interested behavior, whereas others suggest that it will produce more impartial behavior. Here we adjudicate between these perspectives by testing the effects of adopting a third-person perspective on decision making in a task that pits rational self-interest against impartiality: the dictator game. Aggregating across three experiments (*N* = 774), participants who used third-person (i.e., distanced) vs. first-person (i.e., immersed) self-talk during the dictator game kept more money for themselves. We discuss these results in light of prior research showing that psychological distance can promote cooperation and fairmindedness and how the effect of psychological distance on moral decision-making may be sensitive to social context.

## Introduction

When LeBron James was 25-years old, he had to make an agonizingly difficult decision: whether he should stay with his current team, the Cleveland Cavaliers, or join the Miami Heat. Being from Akron, Ohio and having been drafted by the Cavaliers, James could have chosen the Cavaliers out of loyalty to his home-state and fairness to the franchise that drafted him. By joining the Heat, though, LeBron would have more talented teammates, an organization with a championship pedigree, warmer weather, and no state income tax. In other words, James had to choose what was best for himself and what was best for the many fans that wanted him to stay in Cleveland.

James ultimately chose to join the Heat, explaining to an interviewer, “I wanted to do what’s best for LeBron James and to do what makes LeBron James happy.”^1^. Notably, by using his own name to refer to himself in the above quotation, James thought about himself from a third-person perspective when making this decision. In this case, this linguistic shift co-occurred with a self-interested decision. But to what extent did James’ choice of language actually help promote his rational self-interest?

### Distanced-self talk

Self-talk is a basic dimension of one’s inner experience, along with other dimensions such as feeling and sensory awareness, and differs from other forms of thought in that it uses language, as opposed to images or unsymbolized thinking^2^. There are also different kinds of self-talk—for instance, some dual-systems models differentiate between spontaneous and goal-directed self-talk^3^.

Relevant to the present research, people often talk about the self using their given name, a phenomenon sometimes referred to as *illeism*^4,5^ or *distanced self-talk*^6,7^. Only recently, however, has research explored the psychological function of this linguistic shift. In particular, research indicates that when people use their own name and other non-first person pronouns (e.g., he/she, you) to refer to the self, they are able to relatively effortlessly transcend their default, egocentric perspective and see the self from a distanced, third-person viewpoint^8^. More importantly, distanced self-talk can facilitate emotion-regulation^9–11^ (for reviews see^7,12^) and elements of wise reasoning (e.g., intellectual humility)^13,14^. Yet, as the LeBron James example indicates, it is possible that this type of distanced self-talk may also have another important function: promoting rational self-interest during decision-making.

### Why self-distancing might promote rational self-interest

Many theories of human judgment and decision-making, from Plato’s writings to contemporary dual-systems models, have suggested that our intuitions can interfere with rational thinking and behavior. Consistent with such accounts, deliberative (i.e., slow and reflective), compared to intuitive (i.e., fast and automatic), thinking is associated with more rationally self-interested economic decisions that adhere closer to normative models of economic behavior^15–19^. Given research linking psychological distance to increased deliberation^20,21^, distanced self-talk could increase rational self-interest by promoting more deliberative thinking.

Similarly, given theorizing that a third-person, “outside view” can promote more rational decision-making^22^, distanced self-talk may also promote more rationally self-interested decisions. Supporting this possibility, people making financial decisions for others (versus the self) make more rationally self-interested decisions that conform to normative models of economic behavior^23,24^. Research also shows that distanced self-talk can increase objectivity in judgments and decisions^13,14,25,26^, albeit in contexts unrelated to economic decisions. Finally, one study found that people use more second-person language during goal-directed (vs. spontaneous) self-talk^27^, suggesting that psychological distance may foster goal-directed (i.e., rational) behavior. Taken together, distanced self-talk may shift people’s thinking to be more rational, increasing rationally self-interested financial decisions.

### Why self-distancing might decrease rational self-interest

Research from other areas suggests, however, that there are instances in which distanced self-talk may decrease rational self-interest by promoting a sense of fairness or impartiality^28^. This line of reasoning can be found in Adam Smith’s treatise on the self and morality, *The Theory of Moral Sentiments.* According to Smith, humans are naturally self-centered, but that if we imagine ourselves “at a certain distance…from the place and with the eyes of a third person,” we are able to see “the real littleness of ourselves” and to make a “proper comparison between our own interests and those of other people.” In other words, Smith believed that a distanced view of the self would allow people weigh their interests and those of other people less partially and to behave more fairly. Although research has yet to test the full extent of Smith’s logic, several of the underlying principles have been empirically demonstrated.

For instance, research shows that distanced self-talk promotes impartiality. In one set of studies, self-distanced (vs. self-immersed) reflection was associated with less extreme political ideology and greater willingness to join bipartisan discussion groups^14^. In another set of studies where participants imagined close others having committed crimes, participants using distanced (vs. immersed) self-talk were less likely to lie on behalf of their felonious close others to protect them from punishment, demonstrating that distanced self-talk can promote fair-minded (e.g., honest) and impartial (i.e., nondiscriminatory) behavior^25^. Finally, one study demonstrated that distanced self-talk was associated with increased cooperative behavior in a public goods game, which involves players privately choosing how much money to contribute to a shared pot, with the pot’s sum multiplied and distributed evenly among the players^29^.

### The present research

Critically, prior research cannot clearly distinguish whether distanced self-talk is more likely to promote rational self-interest or fair-minded impartiality because the two outcomes are not at odds with one another in prior research. For instance, cooperation in a public goods game has the potential to benefit the self; likewise, lying to a police officer to protect a criminal loved one can jeopardize one’s own reputation or innocence.

Thus, we aimed to test whether distanced self-talk increases rational self-interest in a situation where rational self-interest is at odds with fair-minded impartiality: the dictator game^30^. In the dictator game, participants are randomly assigned to the role of “decider” or “recipient.” The decider is allotted a sum of money and can choose how much money to give (if any) to the recipient. Unlike in other economic games (e.g., a public goods game, an ultimatum game), there is no financial incentive to give money to the recipient in the dictator game, and in fact, giving money to the recipient always comes at a financial cost to the self. Thus, the dictator game is a situation where rational self-interest or fair-minded impartiality are not conflated with one another.

In the present research, we conducted three experiments with samples from two populations (university undergraduates and participants from Amazon’s Mechanical Turk) to examine the effect of self-distancing on rational self-interest. In each study, participants played the dictator game as the decider, but were randomly assigned to refer to themselves in either first or third-person language while thinking about their decision (i.e., immersed vs. distanced self-talk^9^).

The first experiment (Study 1) recruited university undergraduates in an exploratory study testing the two competing predictions pertaining to the relationship between self-distance and rational self-interest. The second experiment (Study 2) sought to replicate the first study with a sample from a different population (Amazon’s Mechanical Turk). The third experiment (Study 3) was mostly identical, except that we had participants write about their decision while referring to themselves in either first or third-person language. We did this so that we could get a window into participants’ thinking, and whether they were thinking about their decision in different ways across conditions. In particular, we coded the degree to which participants were thinking about the self, others, and moral principles (e.g., fairness), given that each of these themes may be tied to how much money participants keep for the self (vs. give to others) in the dictator game. Results focus on data collapsed across all studies given the similar designs and outcomes.

## Results

We tested whether distanced self-talk affects rational self-interest for each individual study and meta-analytically by collapsing data across studies. We first present these results. Following this, we analyze results from the coded essays in Study 3 to examine if the effects of distanced self-talk on rational self-interest could be explained by shifts in how participants thought about the decision.

### Does distanced self-talk influence rational self-interest?

We tested whether distanced self-talk influenced rational self-interest using an ANOVA that included “condition” (distanced vs. immersed), “study” (Study 1 vs. Study 2. vs. Study 3) as categorical covariate, and the condition x study interaction. For this ANOVA, the dependent variable for Study 3 was doubled so that it used the same scale as Studies 1 and 2 (i.e., out of $10). In a separate model, we ran this ANOVA, but without the condition by study interaction term included—the effect of condition was the same across both studies. Below, we report results for this ANOVA with the interaction term for completeness, including estimated marginal means to measure central tendency and their 95% confidence intervals to estimate variance.

Across studies, participants using distanced self-talk to think through their decision kept more for themselves (*M* = $7.30; 73.0% of the money) than those in the self-immersed condition (*M* = $6.78; 67.8% of the money), *F*(1, 768) = 7.83, *p* = 0.005, *d* = 0.20. There was also a main effect of study (*F*(2, 768) = 29.84, *p* < 0.001, η^2^_p_ = 0.072), which captured that MTurk participants kept more money for themselves than those from the subject pool (see in Table 1). The condition x study interaction was not significant, *F*(2, 768) = 1.83, *p* = 0.16, η^2^_p_ = 0.005.

**Table 1:** Effect of self-distancing on money kept by participants.

	Immersed *M (SD)*	Distanced *M (SD)*	Inferential statistics
Study 1	5.54 [1.80]	6.28 [2.41]	*t*(139) = 2.01,* p* = .04*, d* = .35
Study 2	7.38 [2.45]	7.46 [2.34]	*t*(396) = 0.36,* p* = .72*, d* = .04
Study 3	7.43 [2.52]	8.17 [2.22]	*t*(233) = 2.27,* p* = .02*, d* = .31

In addition, we analyzed the effect of distanced self-talk in each individual study. Participants using distanced (vs. immersed) self-talk to think about their decision condition kept significantly more money for themselves in Study 1 and Study 3; there was no difference between conditions in Study 2. See Table 1 for raw means and standard deviations across conditions and independent t-tests for each study.

Finally, given that our observed effect size (d = 0.20) was smaller than what we initially predicted (d = 0.50), we wanted to run a post-hoc power-analyses to estimate how much power we had to observe the effect of interest in the aggregated analysis and in each individual study. Using G*Power ^31^, a post-hoc power analysis (using two-tails, α = 0.05) revealed that we had sufficient power (power = 0.80) to detect the aggregate effect size (d = 0.20) in the aggregated sample using an ANOVA (as was used in the present analyses), and that we were underpowered to detect the this effect in Study 1 (power = 0.22), Study 2 (power = 0.51), and Study 3 (power = 0.33).

### What mediates the relationship between distanced self-talk and rational self-interest?

**Figure 1:**
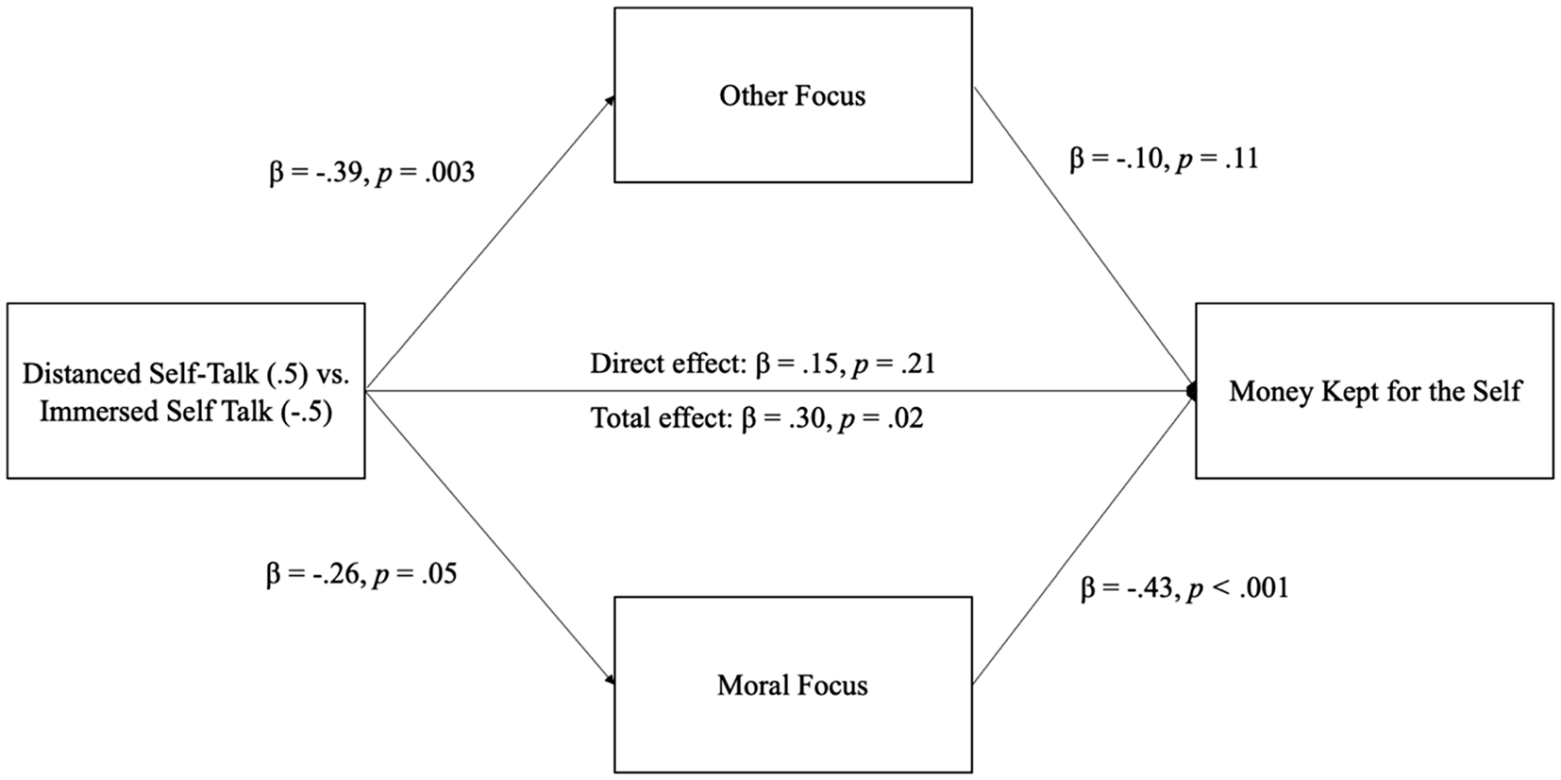
Mediation model from Study 3. Participants using distanced (vs. immersed) self-talk thought less about other people and less about moral principles. Decreases in moral-focus (but not other-focus) mediated the finding that distanced (vs. immersed) self-talk was associated with more money kept for the self. All β values represent partially standardized coefficients, consistent with recommendations from Hayes (2017) to use partially standardized coefficients with dichotomous X variables.

We tested other-focus and moral-focus as simultaneous mediators in a single model (Model 4) using the PROCESS Macro ^32^; we did not test self-focus as a potential mediator because it was not affected by condition (*t*(233) = 0.65, *p* = 0.52, *d* = 0.09) . Results revealed a significant indirect effect through moral-focus (partially standardized indirect effect = 0.11, 95% CI [0.003, 0.236], SE = 0.06, 37.3% of the total effect), but no indirect effect through other-focus (partially standardized indirect effect = 0.04, 95% CI [− 0.01, 0.12], SE = 0.03, 12.7% of the total effect). In addition, the direct effect of condition on rational self-interest was no longer significant after accounting for these two indirect effects (unstandardized effect = 0.37, 95% CI [− 0.21, 0.95], SE = 0.30, *p* = 0.21). See Fig. 1.

## General discussion

How does adopting an objective, third-person view of the self influence rational self-interest? For basketball superstar LeBron James, his use of distanced self-talk famously coincided with the difficult, but rationally self-interested decision to switch teams during free agency. Prior research, however, offered mixed forecasts as to whether a distanced perspective of the self would promote rational self-interest vs. fair-minded impartiality when the two were at odds with one another. The present research offers insight into this debate by examining how distanced self-talk affects decision-making in a zero-sum game where rational self-interest and impartiality are at odds with one another. Aggregating across three studies, participants using distanced (vs. immersed) self-talk kept more money for themselves in one-shot, anonymous dictator games, suggesting that distanced self-talk promotes rational self-interest. This effect was observed among college students in a laboratory setting, as well as among MTurk participants in an online setting. The aggregated effect size (*d* = 0.20) is small by conventional standards, but is comparable to the estimated effects from several dictator game meta-analyses, including trait altruism (*d* = 0.22; ^33^); gender (*d* = 0.25; ^34^); the presence of eye-like stimuli prompting donations (*d* = 0.18; ^35^); and stake size (*d* = 0.15; ^36^).

### Theoretical implications

Findings from the present research accord with other research showing that distanced self-talk promotes rational thinking and behavior^26^. The economically rational decision in this context—a one-shot, anonymous allocation decision—is to keep money for the self, given that there is no opportunity for retaliation, reputation costs/benefits, or reciprocity. Defining the decision to keep money as rational is consistent with normative economic (i.e., maximizing expected utility;^37^) and evolutionary (acquiring resources that increase biological and reproductive fitness;^38^) conceptualizations of rational behavior.

This broader trend for self-distancing to promote rational thinking and behavior may explain seemingly contradictory findings in other research, in which distanced self-talk was associated with increased money given to others in a different economic game—a public goods game^29^. Unlike the one-shot dictator game used in the present research, a public goods game offers the possibility for cooperation to result in increased gains for the self, meaning that cooperative behavior can be consistent with rational self-interest. Thus, it is possible that distanced self-talk allows one to behave in the way that is more rational and beneficial for the situation at hand (i.e., keeping money in a one-shot dictator game, cooperating in a public goods game), consistent with the idea that deliberation will only increase selfish decisions when it is the most beneficial option^39^. Future research should more systematically examine how these contextual variables modulate the effect of self-distancing on people’s moral cognition and behavior.

These findings are consistent with research on construal level theory, i.e., the idea that psychological distance can result in more abstract and decontextualized construals of people, objects, and situations^40^. Prior research on construal level theory has shown that other dimensions of psychological distance (e.g., social distance; temporal distance) and abstract thinking can shift people’s economic decisions to be more in line with their future or desired selves, such as reducing preferences for smaller present rewards over greater future rewards (i.e., temporal discounting)^41–43^ and rejections of unfair but objectively beneficial financial offers^44^. The fact that self-distance may have the similar effects on rationally self-interested decision-making as other dimensions of psychological distance (e.g., temporal distance; social distance) is consistent with research showing that different dimensions of psychological distance have similar psychological and behavioral consequences^45^.

The present work also accords with prior work examining the effect of delegation on allocation decisions in the dictator game. When dictators delegate allocation decisions to third parties, they choose third parties that will allocate less money to the recipients and more money to themselves. In other words, these delegates function as a buffer between dictators and recipients, warding off concerns people feel about appearing selfish^46^. In the present research, distanced self-talk may have had a similar effect, shifting the focus from “How much money should I give to the other person?” or “What is the fair decision?” to “What is in the best interest of the person making this decision (i.e., the self)?” Future research should explore whether there are contexts in which different types of psychological distance (e.g., distanced self-talk vs. making decisions on behalf of others) produce divergent effects in financial decision-making.

### Limitations and future directions

The present research offered a window into how distanced self-talk affects rational self-interest, but there are several limitations to our work that should be considered when interpreting the results.

First, our self-talk instructions may have prompted other kinds of inner experience beyond self-talk, such as inner seeing^2^. This would actually be consistent with other research showing that third-person (vs. first person) self-talk results in people visualizing the self from a more distanced perspective^9^. Thus, it is possible that our first-person vs. third-person manipulation also changed other dimensions of inner experience, and these changes may have also had effects on participants’ dictator game decisions.

Second, our research is limited in that it examined experimentally-induced self-talk, as opposed to spontaneous self-talk. For instance, typical spontaneous self-talk is not prompted by others or written down (as it was in Study 3). Although future research could test the effects of distanced (vs. immersed) spontaneous self-talk on rational self-interest, prior research suggests similar effects would emerge. For instance, prior research on spontaneous *visual* self-distancing shows that, like experientially induced visual self-distancing manipulations, it carries similar emotion regulation benefits (e.g., less rumination).

Third, our individual studies were underpowered to detect the effect size that we observed in the meta-analysis. Therefore, we encourage readers to interpret the results from individual studies with caution, and to instead treat the meta-analysis as the best estimate of the effect of distanced self-talk on rational self-interest.

Fourth, our research was limited by focusing on a single task. As previously discussed, the effects of self-distancing appear to depend on context, and thus, more work is needed to examine whether different tasks and contexts moderate the effects of self-distancing on people’s moral judgments and decisions.

Finally, and related to the above point, it is possible that there are individual differences that moderate the effects of distanced self-talk has different effects on rational self-interest. In particular, given that rationality is often defined as people behaving in line with their own goals and preferences^47^, distanced self-talk may have heterogeneous effects for people with different goals and preferences, with the common thread being the distanced self-talk promoting instrumental rationality.

## Method

### Study 1

#### Participants

Participants were recruited from the University of Michigan Psychology Department’s Paid Subject Pool. They were told that they could earn up to $10 by participating. We anticipated a moderate effect (*d* = 0.50) for self-distancing based on the previous research on self-distancing and decision-making^9,13,25^. We used G*Power^31^ to determine that we needed 128 participants to have 80% power to detect this effect. We recruited 10% more than our target to account for potential exclusions (*N* = 145*)*. Four participants were excluded failing our a priori criteria for quality responses (two had taken similar studies; one did not have a gender-matched experimenter; one was not a native English-speaker), leaving a final sample of 141 participants (59.6% female) with 80% power to detect an effect size of *d* = 0.48.

#### Procedure

Participants arrived at the laboratory at the same time as one other participant of the same gender. An experimenter of the same gender as the two participants told them they would be participating together in the experiment and led them to computers in separate cubicles, where participants completed informed consent and read about the tasks they would be doing. Participants then answered questions about their current emotional state (for descriptive statistics, see Supplementary Table S1). All participants entered their first name into the survey so that it could be piped into later prompts for those in the self-distancing condition. Next, participants were briefed on the rules of the dictator game and told that they and their partner would be assigned to either the role of the *decider* or the *recipient* (in reality, all participants were assigned the role of the decider).

From there, participants were randomly assigned to either the self-immersed or the self-distanced condition. Participants in the self-immersed condition read: “Some people report thinking about themselves using first person language, so that’s what we’d like you to do as you make your decision. In other words, as you think about how to split up the money, ask yourself things like, ‘What should I do?’”.

Participants in the self-distanced condition read: “Some people report thinking about themselves using third person language, so that’s what we’d like you to do as you decide what to do when making your decision. In other words, as you think about how to split up the money, ask yourself things like, “What should [participant’s name] do?’” with the participant’s first name piped into the question from when they entered it earlier. Note that in both conditions, we specifically prompted participants to engage in self-talk (as opposed to other types of thinking) by asking them to think to themselves verbally (the defining feature of self-talk) and providing an example using language, consistent with other research that has used similar instructions to elicit self-talk^9,26^.

All participants then chose to keep anywhere from $10 dollars to $0, with the different options representing one-dollar increments in between those two extremes (participants were given this exact dollar amount at the end of the study). Participants in the self-immersed condition chose from options that used “I” (e.g., “I keep $10 and give the recipient $0), whereas participants in the self-distanced condition chose from options that had their name piped into the text (e.g., “[Name] keeps $10 and gives the recipient $0”). Participants kept an average of $5.89 (*SD* = 2.14) out of a possible $10.

Participants then rated satisfaction with their decision, completed follow-up measure of their emotional state (for descriptive statistics, see Supplementary Table S1) and were funnel-debriefed by the experimenter.

### Study 2

#### Participants

Participants from the United States were recruited from Amazon Mechanical Turk (MTurk). We needed 260 participants to detect the observed effect distanced self-talk in Study 1 (Power = 0.80 for *d* = 0.35 and α = 0.05). We recruited 400 participants to account for potential attrition and the fact that we were sampling from a new population. One participant was excluded for failing to enter their first name and one participant was a research assistant testing the survey, leaving a total of 398 participants (43.5% female; *M*_age_ = 34.4; 80.2% white), giving us 94% power to detect our effect size of interest.

#### Procedure

Participants were told that they would be paid a flat fee of $0.50 but could earn an additional $10 depending on how they performed during the experiment. After consenting to participate, participants completed a measure of baseline mood (see Supplementary Table S1 for descriptive statistics) and entered their first name into the survey so that it could be piped into later prompts.

Participants were then briefed on the rules of the dictator game and told that they would be randomly partnered with another MTurk participant. From there, participants were directed to a screen that had a loading icon and the text, “Please wait while we pair you with another MTurk worker. (Usually less than one minute.).” All participants saw this screen for 17s before the survey automatically advanced to the next page. There, all participants were told they had been assigned to the “Decider” role and reminded of the decision that this role entails.

Next, participants were randomly assigned to either the distanced vs. immersed self-talk condition and subsequently chose how much money to keep for themselves. The instructions used, the manipulation, and the choice phase were identical to Study 1. Participants kept an average of $7.42 (*SD* = 2.39) out of a possible $10.

Participants rated satisfaction with their decision, a follow-up measure of mood akin to the measure of baseline mood (see Supplementary Table S1 for descriptive statistics), a series of personality scales that were intended for exploratory analyses (these were not explored in the present research; see supplementary materials for all scales), and demographics.

### Study 3

#### Participants

We recruited 296 participants from the United States from MTurk. This gave us 85% power for detecting the effect observed in Study 1. Participants in Study 3 were instructed to write about their decision using the language associated with the condition they were placed in (i.e., first-person or third-person language). There were 61 participants who were excluded for failing to do this (all in the distanced self-talk condition). This left a final sample of 235 (40.8% female; M_age_ = 34.64; 74.0% white), giving us 74% power to detect the effect observed in Study 1.

#### Procedure

The procedure in Study 3 was the same in Study 2 except for two differences. First, participants were told that they would be paid a flat fee of $0.50 but could earn an additional $5 depending on how they performed during the experiment (they could earn additional $10 in Study 2). This affected the choice set available to participants (they could choose to keep $5, $4, $3, $2.50, $2, $1, or $0). Participants kept an average of $3.86 (*SD* = 1.22) out of a possible $5. Second, participants wrote about their decision using distanced vs. immersed self-talk (rather than merely thinking in those terms, as in Study 2). Even though participants wrote down their thoughts, we treated these thoughts as self-talk given that they were self-directed and mediated by language, consistent with other research that has analyzed written responses using self-talk frameworks^9,26,48^.

#### Measures

Two condition-blind coders read all essays and coded them for five variables: Self-focus, Other focus, Moral focus, Altruism focus, and Emotionality. “Self focus” was defined as, “To what degree does the participant focus their attention on themselves?” (Cronbach’s α = 0.68). “Other-focus” was defined as, “To what degree does the participant focus their attention on the recipient?” (Cronbach’s α = 0.81). “Moral focus” was defined as, “To what degree does the participant focus on moral considerations (e.g., fairness, equality, rights, right/wrong, ‘shoulds’)?” (Cronbach’s α = 0.84). “Altruism focus” was defined as, “To what degree does the participant focus on altruism, explicitly? By altruism, we aren’t referring to fairness or equality, but rather the specific motivation to help or benefit another person” (Cronbach’s α = 0.59). “Emotionality” was defined as “To what degree to they express or refer to their own emotions? This can be explicit (them writing about their feelings) or something you perceive in their writing (their emotions leaking out more subtly through their writing)” (Cronbach’s α = 0.87).

Each of these variables was coded on a 0 (the concept was not at all mentioned) to 3 (the concept was a major theme in the essay) scale. Descriptive statistics and correlations between all five variables are in Supplementary Tables S2 and S3. We review findings pertaining to “Altruism focus” and “Emotionality” in the supplement, because interrater reliability for “altruism focus” was low (Cronbach’s α = 0.59) and because the authors agreed that the “emotionality” code was an insufficient measure of the emotions that participants were feeling regarding their decision. We proceeded to test whether the first three coded variables mediated the effect of the self-talk manipulation on money kept.

### Ethics declarations

All research protocols was approved by the University of Michigan Institutional Review Board—Health Sciences and Behavioral Sciences and performed in accordance with their guidelines and APA ethical standards. Across studies, we obtained informed consent from all participants.

## Supplementary Information

Supplementary Information.
