# Supplementary material for: Distanced self-talk increases rational self-interest
Source: Sci Rep. 2022 Jan 11;12:511. doi: 10.1038/s41598-021-04010-3 (PMC8752811; doi:10.1038/s41598-021-04010-3)
Supplement: Supplementary file 1 — Supplementary Information. [file 41598_2021_4010_MOESM1_ESM.docx]

**Supplementary materials for “Distanced Self-Talk Increases Rational Self-Interest”**

**Study 1**

**Method**

**Measures and Results**

***Self-assessment mannikin***

Participants used the self-assessment mannikin before and after their decision to rate their current emotional valence (1 = very negative, 9 = very positive) and intensity (1 = not at all intense, 9 = extremely intense) [^1^]. A 2 (condition: immersed vs. distanced) x (2: time 1 vs. time 2) within-subjects ANOVA indicated that condition did not significantly affect valence (*F*(1, 138) = 0.91, *p* = .34, η_p_^2^ = .001) or intensity (*F*(1, 138) = 3.62, *p* = .06, η_p_^2^ = .03). For means and standard deviations see Supplementary Table S1.

Supplementary Table S1

Descriptive stats for affect and mood for all studies

|  |  | | |  | | |  | | |  | | | |  |  |
| --- | --- | --- | --- | --- | --- | --- | --- | --- | --- | --- | --- | --- | --- | --- | --- |
|  | | Immersed | | | Distanced | | |  |  | |  |  |  |  |  |
|  | | *M* | *SD* | | *M* | *SD* | |  |  |  |  |  |  |  |  |
| Study 1 SAM intensity, pre-task | | 3.64 | 1.88 | | 3.38 | 1.47 | |  |  |  |  |  |  |  |  |
| Study 1 SAM valence, pre-task | | 6.45 | 1.48 | | 5.92 | 1.44 | |  |  |  |  |  |  |  |  |
| Study 1 SAM intensity, post-task | | 3.51 | 1.69 | | 3.84 | 1.89 | |  |  |  |  |  |  |  |  |
| Study 1 SAM valence, post-task | | 3.74 | 1.48 | | 6.26 | 1.75 | |  |  |  |  |  |  |  |  |
| Study 2 mood, pre-task | | 3.83 | 0.81 | | 3.87 | 0.85 | |  |  |  |  |  |  |  |  |
| Study 2 mood, post-task | | 4.17 | 0.81 | | 4.14 | 0.92 | |  |  |  |  |  |  |  |  |
| Study 3 mood, pre-task | | 3.89 | 0.77 | | 3.75 | 0.92 | |  |  |  |  |  |  |  |  |
| Study 3 mood, post-task | | 4.14 | 0.82 | | 4.10 | 1.03 | |  |  |  |  |  |  |  |  |

***Decision Satisfaction***

Participants answered, “How satisfied are you with your decision?” using a scale from 1 (Extremely dissatisfied) to 9 (Extremely satisfied). Satisfaction was similar in the self-distanced (*M* = 6.70, *SD* = 2.07) and self-immersed (*M* = 6.96, *SD* = 2.00) conditions, *t*(138) = 0.76, *p* = .45, *d* = 0.13.

**Study 2**

**Method**

**Measures and Results**

***Mood***

Participants completed one item to measure mood, “Please indicate how you are feeling right now from extremely negative to extremely positive,” on a scale from 1 (extremely negative) to 5 (extremely positive). A 2 (condition: immersed vs. distanced) x (2: time 1 vs. time 2) within-subjects ANOVA indicated that condition did not significantly affect mood (*F*(1, 380) = 0.99, *p* = .32, η_p_^2^ = .003).

***Decision Satisfaction***

Participants answered, “How satisfied are you with your decision?” using a scale from 0 (Extremely dissatisfied) to 100 (Extremely satisfied). Satisfaction was similar in the self-distanced (*M* = 87.15, *SD* = 18.26) and self-immersed (*M* = 86.68, *SD* = 16.72) conditions, *t*(395) = 0.76, *p* = .79, *d* = 0.03.

***Fixed vs. growth mindset for intelligence***

Participants rated their agreement (1 = Strongly agree, 6 = Strongly disagree) with six items (e.g., “You have a certain amount of intelligence, and you can’t really do much to change it”) to measure their fixed vs. growth mindset for intelligence [^2^].

***Independence vs. Interdependent self-construal***

Participants rated their agreement (1 = Strongly disagree, 7 = Strongly agree) with twenty items (e.g., “I always try to have my own opinions”) to measure their relative independence vs. interdependence [^3^].

***Social values orientation***

Participants answer six items about hypothetical monetary distributions to measure individual differences in social value orientation (i.e., altruistic, prosocial, individualistic competitive) [^4^].

***Moral identity***

Participants rated their agreement (1 = Strongly disagree, 5 = Strongly agree) with 13 items (e.g., “It would make me feel good to be a person who has these characteristics”) to measure their identity [^5^].

***Big Five Inventory***

Participants rated their agreement (1 = Strongly disagree, 7 = Strongly agree) with 10 items (e.g., “I see myself as: extroverted, enthusiastic”) to measure their personality [^6^].

**Study 3**

**Method**

**Measures**

The scales used in Study 3 were the same as in Study 2, except that we removed the measures of intelligence mindsets and the social values orientation, and added the Bem sex-role inventory [^7^]. For this, participants were asked, “Please rate how much each attribute describes who you think you are using the scale below” on a scale from 1 (doesn't describe me at all) to 7 (describes me very well) and subsequently rated themselves on 60 attributes.

**Results**

**Mood**

Mood did not differ between conditions. A 2 (condition: immersed vs. distanced) x (2: time 1 vs. time 2) within-subjects ANOVA indicated that condition did not significantly affect mood (*F*(1, 222) = 0.78, *p* = .38, η_p_^2^ = .004).

**Satisfaction**

Satisfaction was similar in the self-distanced (*M* = 82.73, *SD* = 22.15) and self-immersed (*M* = 85.49, *SD* = 18.61) conditions, *t*(233) = 0.76, *p* = .31, *d* = 0.14.

**Coded variables**

Distanced self-talk affected Other focus and Moral focus, but not the other three variables that were coded from the free response essays about their decisions (see Supplementary Table S2). For correlations among these variables (including money kept for the self), see Supplementary Table S3.

| Supplementary Table S2  Effect of self-distancing on coded variables from Study 3 essays | | | |
| --- | --- | --- | --- |
| Coded Measure | Immersed *M (SD)* | Distanced *M (SD)* | Inferential Statistics |
| Self focus | 1.61 (0.80) | 1.54 (0.82) | *t*(233) = 0.65, *p* = .52, *d* = 0.09 |
| Other focus | 1.13 (0.82) | 0.80 (0.80) | *t*(233) = 2.98, *p* = .003, *d* = 0.40 |
| Moral focus | 1.25 (1.16) | 0.95 (1.06) | *t*(233) = 1.97, *p* = .05, *d* = 0.27 |
| Altruism focus | 0.23 (0.48) | 0.13 (0.42) | *t*(233) = 1.47, *p* = .14, *d* = 0.20 |
| Emotionality | 0.51 (0.74) | 0.47 (0.83) | *t*(233) =0.42, *p* = .67, *d* = 0.06 |

Supplementary Table S3

Correlations among coded variables from Study 3 essays and money kept in dictator game

|  |  | Money kept | Self focus | Other focus | Moral focus | Altruism focus | Emotionality |
| --- | --- | --- | --- | --- | --- | --- | --- |
| Money kept | r | — |  |  |  |  |  |
|  | p-value | — |  |  |  |  |  |
|  |  |  |  |  |  |  |  |
| Self focus | r | 0.42 | — |  |  |  |  |
|  | p-value | < .001 | — |  |  |  |  |
|  |  |  |  |  |  |  |  |
| Other focus | r | -0.21 | -0.27 | — |  |  |  |
|  | p-value | 0.001 | < .001 | — |  |  |  |
|  |  |  |  |  |  |  |  |
| Moral focus | r | -0.46 | -0.24 | 0.23 | — |  |  |
|  | p-value | < .001 | < .001 | < .001 | — |  |  |
|  |  |  |  |  |  |  |  |
| Altruism focus | r | -0.31 | -0.07 | 0.41 | 0.18 | — |  |
|  | p-value | < .001 | 0.257 | < .001 | 0.006 | — |  |
|  |  |  |  |  |  |  |  |
| Emotionality | r | 0.24 | 0.40 | 0.07 | -0.06 | 0.02 | — |
|  | p-value | < .001 | < .001 | 0.286 | 0.358 | 0.746 | — |

**References**

1. Bradley, M. & Lang, P. Measuring emotion: The self-assessment manikin and the semantic differential. *J. Behav. Ther. Exp. Psychiatry* **25**, 49–59 (1994).

2. Dweck, C. S., Chiu, C. & Hong, Y. Implicit Theories and Their Role in Judgments and Reactions: A Word From Two Perspectives. *Psychol. Inq.* **6**, 267–285 (1995).

3. Singelis, T. M. The Measurement of Independent and Interdependent Self-Construals. *Pers. Soc. Psychol. Bull.* **20**, 580–591 (1994).

4. Murphy, R. O., Ackermann, K. A. & Handgraaf, M. *Measuring Social Value Orientation*. http://papers.ssrn.com/abstract=1804189 (2011) doi:10.2139/ssrn.1804189.

5. Aquino, K. & Reed II, A. The self-importance of moral identity. *J. Pers. Soc. Psychol.* **83**, 1423–1440 (2002).

6. Rammstedt, B. & John, O. P. Measuring personality in one minute or less: A 10-item short version of the Big Five Inventory in English and German. *J. Res. Personal.* **41**, 203–212 (2007).

7. Bem, S. L. The measurement of psychological androgyny. *J. Consult. Clin. Psychol.* **42**, 155–162 (1974).
